# Marine protected areas and children’s dietary diversity in the Philippines

**DOI:** 10.1007/s11111-015-0240-9

**Published:** 2015-06-04

**Authors:** Soumya Alva, Kiersten Johnson, Anila Jacob, Heather D’Agnes, Richard Mantovani, Thea Evans

**Affiliations:** John Snow Inc., 1616 N Fort Myer Dr., 16th Floor, Rosslyn, VA 22209 USA; Westat, 1600 Research Boulevard, Rockville, MD 20850 USA; ICF International, 1725 I St NW #1000, Washington, DC 20006 USA; U.S. Agency for International Development, Ronald Reagan Building, 1300 Pennsylvania Ave, Washington, DC 20523 USA; ICF International, 9300 Lee Hwy, Fairfax, VA 22031 USA; ICF International, 530 Gaither Rd, Rockville, MD 20850 USA; The DHS Program, Blue Raster, 2200 Wilson Blvd., Suite 210, Arlington, VA 22201 USA

**Keywords:** Marine protected areas, Biodiversity, Dietary diversity, Nutrition, Children, Philippines

## Abstract

Fish living around the coral reefs in the Philippines provide livelihoods for more than a million local fishers and are an important source of protein for coastal communities. However, this rich resource is at risk from myriad threats, which consequently threaten human livelihoods, nutrition, and health. In this paper, we examine the degree to which marine protected areas (MPAs), which aim to conserve marine biodiversity, are associated with improved nutritional outcomes in children under age 5. This analysis, which uses data from the 2008 Philippines Demographic and Health Survey and MPA data from the Coastal Conservation and Education Fund, found a positive association between MPAs and children’s dietary diversity when the MPAs were located closer than 2 km to a child’s community. MPA characteristics such as age or type of management were not consistently associated with dietary diversity. These results suggest a positive association of proximity to MPAs with certain aspects of children’s diet.

**Electronic supplementary material:**

The online version of this article (doi:10.1007/s11111-015-0240-9) contains supplementary material, which is available to authorized users.

## Introduction

Coral reefs have the highest biodiversity of any marine ecosystem and provide critically important ecosystem services for coastal communities in the Philippines. It is estimated that fish living around the coral reefs in the Philippines, which is part of the biodiversity-rich Coral Triangle region, provide livelihoods for more than a million local fishers and are estimated to contribute almost USD 1.5 billion to the economy annually (Green et al. 2011).These fish species are also an important source of protein for coastal communities. In addition, healthy and intact coral reefs provide shoreline communities protection from the impacts of natural disasters (Gjertsen 2005).

However, the rich marine biodiversity of the Philippines is at risk from myriad threats including habitat loss, overharvesting of species, pollution, invasive species, destructive fishing practices, and climate change. Of particular concern is the poor state of coral reefs in the Coral Triangle region and in the Philippines specifically. A recent study from the World Resources Institute estimated that more than 90 % of reefs in this region are under threat (Burke et al. 2011).

In recent decades, marine protected areas (MPAs)—“clearly defined geographical space[s], recognized, dedicated, and managed, through legal or other effective means, to achieve the long-term conservation of nature with associated ecosystem services and cultural values” (Laffoley 2008)—have been established worldwide as a means of protecting and restoring degraded marine ecosystems, which provide critically important services, especially for more than 1 billion people globally who rely on fish as their sole source of protein (FAO 2005). MPAs are a tool meant to protect and conserve marine biodiversity, along with critically important ecosystem services, usually through management programs that impose some level of restriction on human activity within the designated area. No-take zones within MPAs offer habitat protection and allow fish populations to mature and reach adulthood, thereby enhancing reproduction and spillover of fish into adjoining areas where fishing is allowed. This can result in increased fish catch for consumption and sale (Gjertsen 2005). In addition, MPAs can also increase revenue for local communities through tourism activities (Lowry et al. 2009). Recognized as effective tools in the sustainable management of marine resources, there are more than 5000 MPAs worldwide (Dudley 2008), with varying levels of effectiveness due to differences in management and enforcement. By conserving ecosystem goods and services, effectively managed MPAs can potentially have a positive effect on the health, nutrition, and livelihoods of nearby human communities.

While the impacts of MPAs on marine ecosystems in the Philippines are generally considered to be positive (Lowry et al. 2009), their effect on the diet and nutritional status of coastal communities is less clear (Mascia et al. 2010). To improve our understanding in this regard, this paper presents the results of an analysis designed to examine the relationship between the presence and characteristics of MPAs and children’s dietary diversity by combining a database of MPAs with data from nationally representative household population, health, and nutrition surveys. We expect the findings to inform policies related to MPAs and contribute to an understanding of the relationship of marine conservation with human health and nutrition outcomes.

### MPAs in the Philippines

Over 1,000 MPAs have been established in the Philippines to date, covering about 15,000 square kilometers (sq. km). They range in size from <1 sq. km to a handful that are more than 1,000 sq. km (Lowry et al. 2009, Weeks et al. 2010). The first MPAs in the Philippines were established as early as the 1970s, with dual objectives: both to restore the coral reefs and fish stocks, and to empower local communities in the management of their resources (White et al. 2002). Early MPAs were shown to have positive impacts on fisheries and coral reef habitats in the areas around Apo and Sumilon islands. As MPAs developed a presence in the Philippines, communities began to realize the negative consequences of unsustainable fishing practices and, concomitantly, the importance of maintaining MPAs (van Beukering et al. 2007). In 1991, national policy changes increased the authority of local coastal municipalities to manage marine resources up to 15 km from the shoreline. This has resulted in the formation of hundreds of locally managed MPAs, and almost 30 national MPAs (White et al. 2002; Green et al. 2011), over the past two decades.

### MPA impacts on biodiversity and the ecosystem in the Philippines

Despite the country’s embrace of the MPA as a conservation measure over the past several decades, the impacts of MPAs on biodiversity conservation are not fully understood. A 2008 analysis of 985 MPAs by Weeks et al. estimated that only about 3 % of coral reefs in the Philippines are within no-take zones, which offer the highest level of protection for biodiversity. While the authors found that MPAs in the Philippines could often meet conservation goals at the local scale, they concluded that “the current extent, distribution, and size of MPAs are inadequate to fulfill conservation objectives at this (national) scale.” Two other recent assessments found that less than one-third of MPAs in the Philippines are meeting their conservation and associated ecosystem services goals (Arceo et al. 2008; Alcala et al. 2008).

With regard to fish species specifically, Maliao et al. (2009) conducted a meta-analysis of nineteen marine reserves, which are the most restrictive of all MPAs in terms of human activity, to determine which characteristics influence fish density in MPAs. The researchers found several characteristics that correlated with increased fish density: how well regulations were enforced, which fish species were being studied, and the age and size of the reserve. Spatial comparison found that older and larger MPAs had higher fish density compared with those that were younger and smaller. Marine reserves also had higher densities of both exploited and non-exploited fish species compared with areas where active fishing was taking place.

Only a few studies have evaluated the impacts of MPAs on ecosystem services that contribute to human health, nutrition, or social outcomes including livelihoods (Mascia and Claus 2009 for example). To address this, researchers have developed a framework to show the relationship between MPA governance and performance, including social impacts on wealth, health, empowerment, and education (Glew et al. 2012). A recent literature review concluded that “MPAs are neither uniformly good nor uniformly bad for coastal communities; rather, the social impacts of MPAs vary within and among groups and subgroups and across different indicators of social well-being” (Mascia et al. 2010). Specifically related to nutrition, the review found that food security for coastal communities generally stayed the same or increased following the establishment of an MPA, although a decline was observed among some fishing subgroups.

**Fig. 1:**
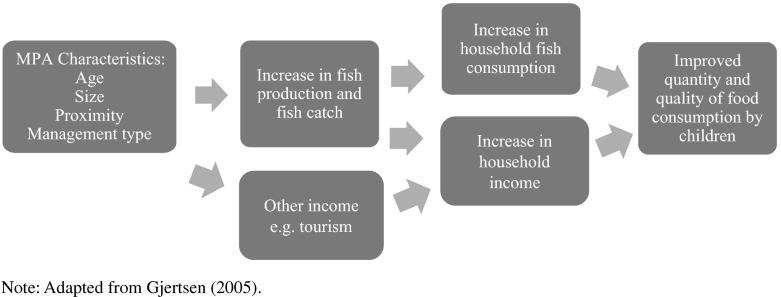
Pathway to improvements in children’s nutrition

In this paper, we use a national database of MPAs and nutrition data from a national survey to examine the relationship between various MPA characteristics (proximity to a child’s community, size, age, and management) and children’s nutrition outcomes following the pathway presented in Fig. 1, which is adapted from Gjertsen’s 2005 study in the Philippines which has a similar hypothesis, focused on the relationship between coral reef health and human well-being as measured by child weight for age in villages close to MPAs in the Philippines. However, unlike Gjertsen’s study, which was restricted to select coastal villages, this research focuses on a national-level analysis of dietary diversity, a more immediate and relatively less complex outcome variable than weight for age.

We hypothesize that proximity to effective MPAs will have a positive effect on children’s dietary diversity as a result of fish abundance. For this hypothesis to be true, we assume that those who live close to an MPA are primarily dependent on marine resources for their income and/or food, particularly as fishers. With greater fish catch as a result of the establishment of an MPA, it is expected that correlated increases in household incomes from sale of fish would in turn result in increased household spending on a more diverse array of foods for family consumption (Leisher et al. 2007). A change in the diet could be a result of other possible mechanisms including livelihood development (e.g., tourism), improved governance, and the strengthening of social networks during MPA establishment and socialization (Fig. 1).

## Data and methods

This analysis uses data from two sources: the Philippines 2008 Demographic and Health Survey (DHS), which provided child nutrition indicators as well as sociodemographic control variables for the analysis; and georeferenced data on MPAs in the Philippines from the CCEF’s MPA database.

### MPA data

The data on MPAs in the Philippines come from the CCEF Marine Protected Coast Reef and Management database, which maintains information on various characteristics of MPAs such as geographic coordinates, size, year of establishment, and an effectiveness rating. We did not use the CCEF effectiveness rating because the majority of the National Integrated Protected Area System (NIPAS) MPAs have not been evaluated. Although restricting the analysis to well-managed MPAs and eliminating the unrated MPAs would be ideal, it would reduce our sample sizes and could bias the analytical results. MPAs that are missing data on latitude/longitude coordinates, the year of establishment, or the size of the MPA are excluded from the analysis. Additionally, the Tañon Strait national MPA is excluded from the study due to the oil exploration activities within the Strait. MPAs that were established after 2008 are also excluded from the analysis, resulting in 526 MPAs included in this analysis. More specific details on the setup of the MPA dataset are presented in Appendix 1.

### DHS data

The DHS are nationally representative household surveys with large sample sizes that provide detailed information on social, demographic, and health indicators, including nutritional status of women and children by interviewing women of reproductive age 15–49 to obtain information on the health, nutrition, and socioeconomic characteristics of the respondents, their children, and other household members. Surveyed households are selected through a two-stage random sampling process. At the first stage, DHS clusters or primary sampling units (PSU) comprised of 100–300 households are identified. In the second stage, a prefixed number of households are selected from a list of households from each PSU. In 2008, the sample of interviewed women includes 13,594 women aged 15–49 residing in 12,469 households; the response rates for both the household and women’s interviews exceeded 99 % (NSO [Philippines] and ICF Macro 2009).

### Linking the DHS and MPA datasets

**Fig. 2:**
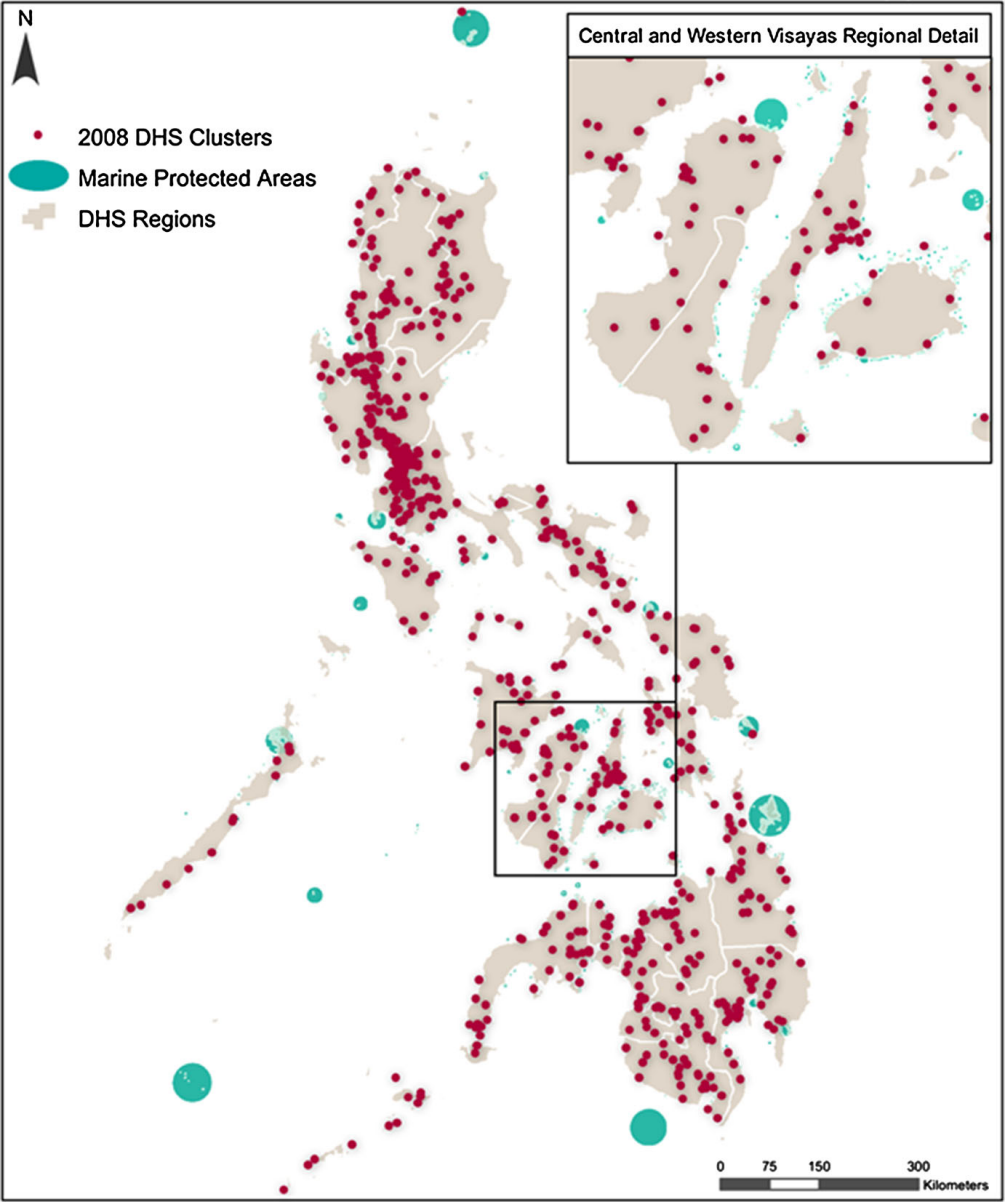
GPS clusters and marine protected areas of the Philippines

**Fig. 3:**
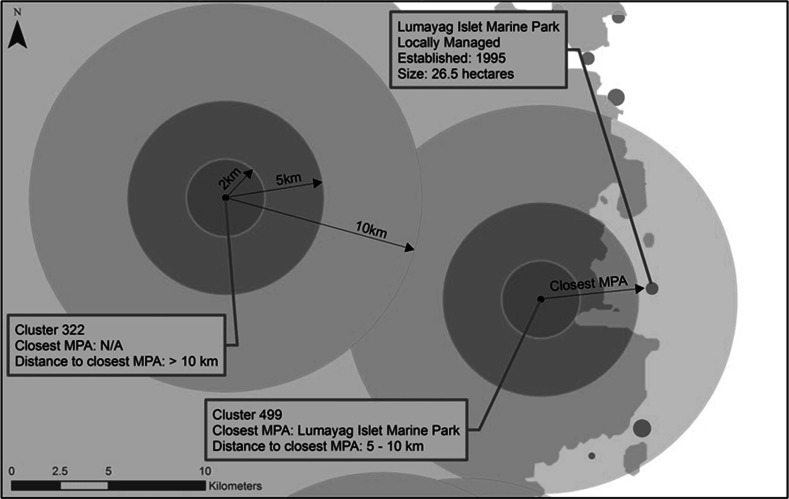
Determining clusters’ proximity to marine protected areas

The DHS data are georeferenced, hence enabling merging with CCEF’s MPA dataset; the DHS data, comprised of 792 sampling clusters, were overlaid with the MPA dataset. The DHS do not georeference each selected household in the sample. Rather, a single point is taken at the approximate geographical center of every DHS sampling cluster; this is the spatial data point for all households within that cluster, based on which proximity to the closest MPA is measured. To further ensure the anonymity of survey respondents, the georeferenced clusters were displaced randomly. Urban clusters contain a minimum of 0 and a maximum of 2 km of positional error. Rural clusters contain a minimum of 0 and a maximum of 5 km of positional error with a further 1 % of the rural clusters displaced a minimum of 0 and a maximum of 10 km. An audit of spatial data collected through the DHS in 2011 showed that on average, DHS clusters in rural areas were displaced <2.5 km from their original location. To better account for the imprecision in the geospatial data resulting from the random displacement of cluster locations, categorical distances were used instead of direct distances. To determine the proximity of MPAs to DHS displaced cluster locations, distances from the displaced cluster to the edge of the closest MPA were calculated and then classified into four categories. In all, 526 MPAs in the Philippines with information on coordinates and size were included in the analysis. The Central Visayas region contains far more MPAs and more DHS clusters close to the coast than any other region in the country. This corresponds with other research on MPAs that show that 67 % of all MPAs are located in the Visayan Sea (Weeks et al. 2010). Figure 2 provides a graphic representation of the DHS clusters, with the MPAs overlaid on the map of the country. Figure 3 demonstrates how the proximity to an MPA was calculated from DHS clusters.

### Study sample

In this analysis, we focus on the dietary consumption of the most recently born child aged 6–59 months born to sampled women. The DHS sampled 5,964 children aged 6–59 months in 2008. Only the most recently born child in the 59 months prior to the survey is included in the analysis to eliminate intra-household correlation. After accounting for missing information on all key and control variables in the regression analysis, the number of children included in the final study sample was 4,382.

### Analysis variables

#### Dependent variable

The outcome of interest in this analysis is children’s minimum dietary diversity. The DHS asked mothers about their young children’s consumption of specific items in various food groups, in the past week and in the past 24 h, for the most recently born child between the ages of 6 and 59 months in the household. We base the calculation of our dependent variable on the WHO definition of minimum dietary diversity: the proportion of children 6–23 months of age who receive foods from four or more of the following food groups: grains, roots, and tubers; legumes and nuts; dairy products (milk, yogurt, cheese); flesh foods (meat, fish, poultry, and liver/organ meats); eggs; vitamin A-rich fruits and vegetables; and other fruits and vegetables (WHO 2007). In this analysis, we measure minimum diversity as a dichotomous variable for the most recent child aged 6–59 months, based on whether he/she received foods from the above-mentioned four or more food groups. We have also adapted the WHO definition of the minimum dietary diversity indicator to cover children aged 6–59 months. A greater number of children are included in our sample because of this increase in age range. All the same, the anticipated relationships examined in this study are expected to remain the same for this broader group of young children.

#### Key independent variables

This analysis examines the relationship of MPA characteristics to dietary diversity using four different characteristics: (1) the categorical distance in kilometers of the index child’s community (sampling cluster) to the closest MPA; (2) the age of the MPA in years, based on the year of establishment; (3) the size of the MPA in hectares; and (4) whether the MPA is locally managed.

For all four MPA-related variables, all MPAs at a distance >10 km from the child’s community are treated as the reference group; 88 % of all MPAs in the analysis sample were at a distance >10 km from the child’s community.^1^ These MPAs are considered too far, and the clusters are coded as “no MPA close by” in the analysis. We expect that communities living <10 km from an MPA are relatively more likely to be dependent upon the coastal and marine resources for their incomes—whether they are fully dependent (such as fishers) or only partially dependent (such as those who collect shells and sea urchins or harvest seaweed) to supplement their incomes and/or diets. The only exception to this could be large coastal cities <10 km from the coast, such as Cebu, where the majority of the population is not likely to be directly dependent on the marine environment for their incomes. The categorical breakdown of the proximity to MPA variable is as follows: <2 km, 2–5 km, 5.1–10 km, and >10 km (no MPA close by).

The age of the MPA is categorized as follows: <3 years, 3–5 years, 6 or more years, no MPA close by. Although these categories were developed based on when the MPA was established, they reflect aspects of the Management Effectiveness Rating System that CCEF developed as part of their MPA database where age of the MPA is a key criteria in the definition of the rating system. According to this classification, MPAs <1 year old (Level 1) are treated as initiated MPAs, with little to no difference between MPA and no MPA. MPAs 1–2 years old are established MPAs which are expected to be managed and somewhat enforced (Level 2). MPAs 3–5 years old are those whose management is enforced and sustained (Levels 3 and 4). MPAs in existence for more than 5 years are expected to be institutionalized (Level 5). Because a large number of MPAs are located at a distance >10 km from households sampled by the DHS, thus falling into the reference group, the number of MPAs in each of the above-specified levels is small. To ensure a sufficiently large number of cases per categorical grouping, the first two categories covering new or recently established MPAs (Level 1 and Level 2) were collapsed, thus grouping all MPAs <3 years old into one category in the analysis.

The other variables of interest include the proximity of the child’s community to the ocean (measured in km and serving as a control variable); child’s sex and relative household wealth status, which is an asset-based index that classifies the sample into five wealth quintiles; mother’s education; and number of children in the household. More specific details on the variables and the grouping for the categorical variables are presented in Appendix 2.

### Statistical analysis method

Broadly based on the pathway presented in Fig. 1, this analysis examines the relationship between MPA characteristics and food consumption of young children as measured by their dietary diversity. Since the outcome variable is a dichotomous variable indicating whether the child does or does not have a diverse diet, the analysis uses a weighted logistic regression model based on the survey regression techniques to account for the sampling design of the survey and is of the form

$$ \log {\text{it}}\left({\mathbb{E}}\left[ {Y_{i} \left| {{\mathbf{X}}_{i} }
\right.} \right] \right) = \log {\text{it}}(p_{i} ) = \ln \left(
{\frac{{p_{i} }}{{1 - p_{i} }}} \right) = \beta \cdot
{\mathbf{X}}_{i} $$

All descriptive and regression analyses are conducted using the svy command in the statistical software Stata version 13, and regression results are presented as odds ratios. All results presented in the next section are based on the analytical sample in the regression taking into account missing information in covariates. Multivariate analytical results examining the relationship between MPA characteristics and minimum dietary diversity and also controlling for child and household characteristics are presented in Table 4. Models 1 and 2 examine the effect of proximity to MPA with (1) no controls, and (2) additional controls for child and household characteristics: child’s sex, mother’s education, number of children in the household, and household economic status. Similarly, Models 3–4 examine the effect of the number of years of establishment of the MPA, models 5–6 examine the effect of MPA size, and Models 7–8 examine the effect of management authority (locally managed vs. not locally managed). Each set of models examines the relationship of a single independent variable with the outcome of interest, with and without controls for proximity to ocean and child and household characteristics.

### Limitations of the study

In understanding the results of this analysis, it is important to consider some of the limitations of the study. Calculations based on both the DHS and MPA data were based on several assumptions and involved approximations. While data on the precise dimensions of national MPAs were available through the CCEF database, this information was not consistently available for locally managed MPAs. Thus, the decision was made to define the dimensions of all locally managed MPAs with a calculation based on the size of the MPA in hectares (available in the CCEF database) and the estimated radius.

In addition to assumptions regarding MPA dimensions, the displacement of DHS clusters also affects the calculation of proximity to the MPA. Recognizing this, the analysis uses a categorical variable for proximity to the MPA with the smallest category not <2 km. It is possible that some households in the smallest category are in reality at a somewhat greater distance from MPAs, thus affecting the relationship with children’s nutritional outcomes. However, it is hard to know to what extent this is the case—a fact that highlights the difficulties displacement poses for spatial analyses of the DHS data. Finally, the relatively small number of children residing near the coast and proximate to an MPA must be acknowledged and caution therefore advised in interpreting the results of the analysis.

Another issue to consider is the fact that there may be some built-in biases around areas with MPAs which cannot be measured in an analytical model. It is possible that households proximate to MPAs are exceptional in that they have the foresight to protect their areas. These communities may have access to greater resources, better Local Government Units, or even better coral reefs. Access to all of these factors may independently influence households’ and children’s nutrition.

## Results

Weighted percent distributions of the four MPA characteristics used in the analysis are presented in Table 1. Close to 90 % of all children in the dataset are located at proximity of 10 or more km from the closest MPA. The number of children living within 2 km of an MPA is 97.^2^ The number of children in the analyses of age and size of MPAs that are <10 km from a child’s community is 837. The mean proximity of sampled households to the ocean is also >15 km, indicating that a majority of households in the sample are not located on the coast.

**Table 1:** Weighted percent distributions of categorical independent variables used in the analysis, based on the most recently born child aged 6–59 months, Philippines 2008 Demographic and Health Survey

Variables	Philippines 2008
MPA characteristics	
Proximity to MPA	
≤2 km	2.0
2–5 km	3.2
5–10 km	6.3
>10 km (No MPA close by)	88.5
Age of MPA	
<3 years	1.3
3–5 years	1.7
6 or more years	8.5
No MPA close by	88.5
Size of MPA	
<10 ha	2.9
10–20 ha	2.7
20–50 ha	2.1
50 + ha	3.8
No MPA close by	88.5
Whether MPA is local	
Locally managed	9.3
Not locally managed	2.1
No MPA close by	88.5
Child and household characteristics	
Proximity to ocean (mean distance in km)	16.4
Child is female	52.5
Mother’s education	
No education	1.7
Primary education	24.7
Secondary education	48.8
Higher education	24.9
Number of children in the household	
<2 children	31.2
2 children	44.0
3 children	21.3
4 or more children	3.6
Household wealth status	
Poorest	28.1
Poorer	23.6
Middle	19.3
Wealthier	17.0
Wealthiest	12.1
*N*	4,382

Table 2 shows descriptive data for children’s food consumption in the past 24 h. Fifty-eight percent of children in the Philippines had a diverse diet. More than 80 % of children consumed grains, while the next most commonly consumed food group was vitamin A-rich foods, with 64 % of children having consumed them in the past 24 h. A little more than 45 % of children were reported by their mothers as having eaten meat and fish, respectively, in the past 24 h. Half the children were reported as having consumed other fruits.

**Table 2:** Weighted percentages of the most recently born child aged 6–59 months with a diverse diet (consumption of 4 + food groups): Philippines 2008 Demographic and Health Survey

Variables	Philippines 2008
Percent with a diverse diet	58.5
Percent consuming	
Grains	83.5
Legumes	15.8
Dairy	55.2
Meat	46.4
Fish	45.4
Vitamin A-rich fruits	63.7
Other fruits	50.1
*N*	4,382

Table 3 shows the relationship between children’s dietary diversity and the various characteristics of the closest MPA situated within 10 km of children’s households: proximity to MPA, age of MPA, size of MPA, and whether the MPA is locally managed. Disaggregating age, size, and management type according to distance from MPA resulted in small numbers of children in each subcategory; it was therefore determined to assess the association between these MPA characteristics and children’s dietary diversity for all children living within 10 km of an MPA, relative to those living at a distance >10 km. In terms of distance to MPA, we found that children living within 2 km of an MPA had the highest levels of dietary diversity, at 79 %, while those living farther from an MPA had observably lower levels of dietary diversity (55–59 %). This difference in dietary diversity is statistically significant and is consistent with our hypothesis.

**Table 3:** Minimum dietary diversity of the most recently born child aged 6–59 months according to the characteristics of the closest MPA: Philippines 2008 Demographic and Health Survey

Variables	Philippines 2008
MPA characteristics	
Proximity to MPA	
≤2 km	78.6
2–5 km	55.3
5–10 km	59.4
>10 km (No MPA close by)	58.5
Age of MPA	
<3 years	57.3
3–5 years	70.5
6 or more years	60.4
No MPA close by	58.5
Size of MPA	
<10 ha	66.0
10–20 ha	62.5
20–50 ha	51.5
50 + ha	63.1
No MPA close by	58.5
Whether MPA is local	
Locally managed	60.5
Not locally managed	65.9
No MPA close by	58.5
*N*	4,382

**Fig. 4:**
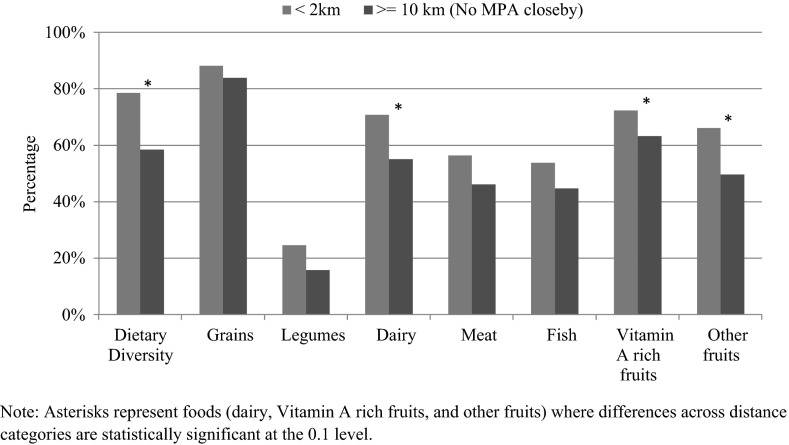
Diet of most recently born child aged 6–59 months by proximity to MPA: Philippines 2008 Demographic and Health Survey

We also examined children’s dietary patterns with respect to each of the seven food groups and distance to an MPA (Fig. 4). The results show consistently higher consumption of each of the component of the diet (grains, legumes, dairy, meat, fish, vitamin A-rich foods, and other fruits) with statistically significant differences by proximity to MPA for overall dietary diversity, dairy, Vitamin A-rich fruits, and other fruits.

There is no clear pattern of association between children’s dietary diversity and the age of the MPA (Table 3). Children living within 10 km of MPAs that had been established for <3 years had the same level of dietary diversity as children living within 10 km of an MPA that was 6 years old or older, about 57–60 %, while children living within 10 km of an MPA that had been in place for 3–5 years had a higher level of dietary diversity, at 70 %. The pattern observed in the relationship between children’s dietary diversity and MPA size is also somewhat unexpected: children’s dietary diversity is inversely associated with size of MPA for the three MPA size categories below 50 hectares (ha); however, the largest MPA size category in this table (≥50 ha) deviates from the pattern. In terms of management of MPAs, the data show that MPAs that are nationally managed are found to have a slightly stronger association with children’s dietary diversity (66 %) but this is not significantly different than the levels of dietary diversity among children living within 10 km of a locally managed MPA (60 %).

Using the non-proximity of MPAs (MPAs at a distance >10 km) from the child’s household as the reference group, the strong effect of distance to the MPA is clearly evident (Table 4). This effect is strong even after controlling for all variables including household wealth, but only for MPAs within a 2-km proximity of the child’s community. For this group, the odds of having a diverse diet are two times more than that of households that are located more than 10 km from MPAs. However, there is little difference between those living at a distance between 2–5 or 5.1–10 km from MPAs as compared to the reference group. With regard to MPA age, Models 3–4 reflect the same association observed in the bivariate analysis: MPAs that were in existence for 3–5 years have the strongest association with children’s dietary diversity.

**Table 4:** Logistic regression results: odds ratios representing the relationship between MPA characteristics and minimum dietary diversity of the most recently born child aged 6–59 months: Philippines 2008 Demographic and Health Survey

	Model 1	Model 2	Model 3	Model 4	Model 5	Model 6	Model 7	Model 8
MPA characteristics								
Proximity to MPA (ref: >10 km)								
≤2 km	2.42**	2.44**						
2–5 km	0.90	0.83						
5–10 km	1.01	1.16						
Age of MPA (ref: >10 km)								
<3 years			0.96	0.82				
3–5 years			1.73^+^	1.86^+^				
6 or more years			1.05	1.15				
Size of MPA (ref: >10 km)								
<10 ha					1.20	1.23		
10–20 ha					0.98	1.07		
20–50 ha					0.67	0.63		
50 + ha					1.24	1.17		
Whether MPA is local (ref: >10 km)								
Locally managed							1.07	1.14
Not locally managed							1.34	1.42
Proximity to ocean (km)		1.00		1.00		1.00		1.00
*N*	4,382	4,382	4,382	4,382	4,382	4,382	4,382	4,382

Models: (1) proximity to MPA with no controls; (2) proximity to MPA with controls for proximity to ocean and child and household characteristics; (3) age of MPA with no controls; (4) age of MPA with controls for proximity to ocean and child and household characteristics; (5) size of MPA with no controls; (6) size of MPA with controls for proximity to ocean and child and household characteristics; (7) management of MPA with no controls; (8) management of MPA with controls for proximity to ocean and child and household characteristics

Controls for child and household characteristics are child’s sex, mother’s education, number of children in the household, and household wealth status

^+^ *p* < 0.1; * *p* < 0.05; ** *p* < 0.01; *** *p* < 0.001

There are no consistent, statistically significant associations between MPA size and children’s diet (see Models 5–6), or between management status (locally managed vs. not locally managed) and children’s diet (Models 7–8). No relationship between proximity to the ocean and children’s diet was observed.

Coefficients for the child and household control variables included in the models are not presented in the tables, but are available in the supplement. Those findings reiterate the broader literature on nutrition outcomes demonstrating that household characteristics have a key role to play in influencing dietary diversity. Although, as expected, differences in diet among boys and girls in the Philippines are not significant, mother’s education and household wealth have strong positive effects on dietary diversity, while households with a greater number of children showed reduced diversity in children’s diets. With more children in the household, the household or mother’s ability to ensure better nutrition levels among children, in this case the most recently born child, is compromised.

## Discussion and conclusions

The health of fisheries in the Philippines has declined over the past few decades, raising concerns about potential economic and nutritional impacts on coastal communities (FAO 2005). In order to slow and reverse marine ecosystem degradation, the Philippines embarked on an ambitious program of MPA establishment, starting almost 40 years ago. Although more than 1000 MPAs have been established since the early 1970s, very little research has been conducted on the health and nutritional impacts of these protected areas on coastal communities. This study introduces a novel methodology to examine some of these relationships at a national scale using existing datasets.

The results of this study shed light on the relationship between several MPA characteristics and children’s minimum dietary diversity in the Philippines. Dietary diversity is an important indicator associated both with child nutritional status (Arimond and Ruel 2004) and household-level food security (Hoddinott and Yisehac 2002). Dietary diversity is also often used as an indicator of adequate micronutrient intake from the diet. While infant and child mortality rates have decreased in the Philippines over the last two decades, malnutrition among children is still a significant issue, with stunting affecting more than one-quarter of all Filipino children. Micronutrient deficiency, especially iron, iodine, and vitamin A deficiencies, is also a significant health issue among children in the Philippines (UNICEF 2008). For communities directly reliant on ecosystem services to meet daily needs, ecosystems that are protected are essential to attaining a diverse diet.

MPAs can provide an array of ecosystem services, including more fish for consumption, higher household incomes generated from selling fish, or tourism-related activities, as well as a sense of empowerment among community members. In communities that live close to MPAs, the revenues from increased fish catch could be used to buy an array of foods that increase overall dietary diversity in children. The results from this study suggest that communities that reside close to MPAs derive many of these benefits, resulting in a positive association with their children’s diverse diets. The relationships of other MPA characteristics included in this study with children’s minimum dietary diversity do not follow a consistent pattern; it is likely that effects of characteristics such as MPA age, size, and type of management would only become apparent at closer proximities to MPAs than the 10-km range to which we were constrained for this analysis. It is possible that the historical trends in the way that MPAs were established in the Philippines are confounding these descriptive observations. According to the literature, larger MPAs are typically expected to have a greater positive effect on reef health with consequent positive outcomes for households. However, if they are too large, there is the possibility that they may not be managed effectively, hence not achieving the intended effect (Gjertsen 2005). Further research looking at the relationship between locally managed and nationally managed MPAs, controlling for effectiveness, is also warranted. With little information on the quality and level of enforcement of MPAs included in this analysis and the consequent increase in fish stocks, it is difficult to discern the reason for the observed associations between children’s dietary diversity and MPA size or age.

Our findings demonstrate a positive relationship between children living in communities within 2 km of an MPA and their fish intake, and dietary diversity in general, suggesting that fish catch is associated with both nutritional and economic outcomes. Families living near MPAs benefit from fishing or from other aspects of MPAs which may allow them to invest in more diverse diets for their children. In our study, these benefits did not extend to families living >2 km from an MPA, suggesting that the positive association of MPAs on children’s nutritional and economic status may be most significant in those communities that live closest to the coast. Fishing communities in the Philippines are often directly reliant on marine resources for food and to generate household income; when these resources become degraded, the loss of critical ecosystem services can have devastating effects on community well-being (Department of Environment and Natural Resources, Bureau of Fisheries and Aquatic Resources of the Department of Agriculture, and Department of the Interior and Local Government 2001). The results from this study suggest that MPAs, which are created to more sustainably manage marine resources, are beneficial to local communities through improved childhood nutrition. Further study is needed to better understand the specific mechanisms through which this relationship operates.

### Implications of findings for programs and policy

Because of their proximity to the sea, communities living within 2 km of MPAs are likely to be highly reliant on fishing as their primary source of income. The most recent poverty statistics for the Philippines indicate that nationwide, fishermen have the highest poverty incidence, at 41.4 %, of nine basic sectors (National Statistical Coordination Board, no date). MPAs can not only improve fishermen’s incomes, by increasing their catch and yields, but also have a trickledown effect upon their children, by improving their nutritional outcomes, their overall health, and future well-being. Accordingly, this research demonstrates that MPAs can be a key strategy to improve well-being among vulnerable and impoverished fishing populations in the Philippines and demonstrates that there may be a multiplier effect on their children’s dietary diversity and health.

Regarding the conclusion of Weeks et al. (2010) that MPAs in the Philippines can often meet conservation goals at the local scale but are inadequate at national scale, we hope that these results showing the importance of MPAs for child nutrition will be seen as contributing to the body of evidence supporting the expansion in size and distribution of effectively managed MPAs throughout the Philippines. However, it is key to note that our research does not propose specific guidelines on the size of MPAs and the relationship to its effectiveness. The human benefits of MPAs tend to be very local, supporting the benefits of coral reefs and MPAs at the local rather than the national scale. While the expansion of MPAs in numbers is relevant to meet national goals, there is need to keep their management and planning to the local level.

At the same time, while MPAs may represent a viable strategy to improve household nutrition and socioeconomic outcomes, there is need for more research to identify the specific set of MPA characteristics that contribute to their overall effectiveness in conserving marine resources and influencing coastal communities. Identifying the set of MPA characteristics that most contribute to their overall effectiveness, including the optimal legal and governance structures and design and socialization strategies, will be important for informing policy as well as in the management of current MPAs and the establishment of new ones.

### Implications of findings for future efforts

Both datasets used in the analysis were collected by different entities for different purposes. As a result, almost 90 % of all MPAs included in the study were located at a distance of 10 or more km from DHS clusters where children in the interview sample lived. Furthermore, as a result of the GPS data displacement in the DHS, it is harder to examine the effect of small distances and the proximity to MPAs especially if they are at a distance of <2 km from children’s residence. Even at distances >2 km, the possibility of classification error remains. This research demonstrates the need to take a coordinated, multidisciplinary approach to data collection, so that the appropriate data may be obtained and analyzed both for the explicit purposes of the organization implementing the data collection and for ensuring precise linkages to external but critically complementary datasets.

Such a holistic and integrated approach, in which the health and environmental communities consciously collaborate, is essential both for building the evidence base for the importance of biodiversity conservation for human well-being, as well as for effectively advocating for the conservation of the environment in order to achieve optimal and sustainable outcomes for ecosystems and humans alike.

### Electronic supplementary material

Supplementary material 1 (XLS 35 kb)

## Appendix 1: MPA data setup

The following steps were taken to give dimensions to the point data, which was the format of the MPAs in the CCEF database.

Each MPA record contained the size of the MPA in hectares.

1. The coordinates for each MPA point were assumed to be the centroid of the MPA. If the record included more than one proxy cluster, the centroid of the boundary coordinates was assumed to be the centroid of the MPA. The shape of each MPA was assumed to be circular. Since a majority of the MPAs are quite small in size, we expect that the approximation of MPA shape will not cause too many data quality issues when combining them with the DHS datasets and analyzing in relation to data on child outcomes in the DHS.
2. The buffering tool in ArcMap was run, with the buffer distance for each MPA assigned based upon following equation
3. $\sqrt {\frac{{(A\,{\text{ha}})\left( {\frac{{10^{4} {\text{m}}^{2} }}{\text{ha}}} \right)}}{\pi }} = {\text{radius}}\,{\text{of}}\,{\text{the}}\,{\text{polygon}}\,{\text{in}}\,{\text{meters}}$ where *A* equals the area of the MPA in hectares.
4. The results of the tool produced a circle for each MPA that is equivalent in size to the area listed in the source data set.
5. For MPAs larger than 10,000 ha that only listed one proxy coordinate, the centroid of the MPA was checked against the WRI/WDPA (http://protectedplanet.net/ and http://www.wri.org/publication/reefs-at-risk-revisited#datasets) georeferenced dataset and when necessary, adjustments were made to match the WRI/WDPA dataset.

## Appendix 2

See Table 5.

**Table 5:** Description of variables

Variable name	Definition
Children’s minimum dietary diversity (6–59 months)	Measured as a dichotomous variable—whether the most recent child aged 6–59 months received foods from four or more of the following food groups—grains, roots and tubers; legumes and nuts; dairy products (milk, yoghurt, cheese); flesh foods (meat, fish, poultry, and liver/organ meats); eggs; vitamin A-rich fruits and vegetables; and other fruits and vegetables. This variable is calculated based on the information on consumption in the last week and in the last 24 h. Some adjustments were made to the categories based on the specific questions asked in the survey
*MPA characteristics*	
Proximity of child’s community to MPA (km): Based on the coordinate of displaced cluster of child’s community to the edge of the MPA	<2 km, 2–5 km, 5.1–10 km, >10 km (No MPA close by: reference group)
Age of MPA (years): Based on the number of years of establishment. MPAs established after 2008 were excluded	<3 years, 3–5 years, 6 or more years, No MPA close by (No MPA close by: reference group)
Size of MPA (ha)	<10 ha, 10–19.9 ha, 20–49.9 ha, ≥50 ha, No MPA close by (No MPA close by: reference group)
Management of MPA	Locally managed, NIPAS or nationally managed, No MPA close by (No MPA close by: reference group)
*Child and household characteristics*	
Proximity of child’s community to ocean (km)	Continuous variable representing mean distance in km
Child’s sex	Female, male (male: reference group)
Mother’s education	No education, any primary education, any secondary education, any higher education (no education: reference group)
Number of children in the household	<2 children, 2 children, 3 children, 4 or more children (<2 children: reference group)
Household wealth status	Poorest, poorer, middle, wealthier, wealthiest quintile (poorest quintile: reference group)

## Footnotes

1 We choose 10 km as the cutoff after examining the distance of DHS clusters from the coast. While a shorter distance such as 5 km may be appropriate, there were insufficient DHS clusters at a shorter distance to the coast to enable such an analysis.

2 Although results may need to be interpreted with some caution, these sample sizes are reasonable to conduct analyses.
